# The monoid-now: a category theoretic approach to the structure of phenomenological time-consciousness

**DOI:** 10.3389/fpsyg.2023.1237984

**Published:** 2023-09-05

**Authors:** Shigeru Taguchi, Hayato Saigo

**Affiliations:** ^1^Faculty of Humanities and Human Sciences, Hokkaido University, Sapporo, Japan; ^2^Center for Human Nature, Artificial Intelligence, and Neuroscience, Hokkaido University, Sapporo, Japan; ^3^Faculty of Bio Science, Nagahama Institute of Bio-Science and Technology, Nagahama, Japan

**Keywords:** time, consciousness, phenomenology, category theory, monoid, Husserl, time-consciousness, meditation

## Abstract

Human consciousness is characterized by constant transitions in time. On the other hand, what is consciously experienced always possesses the temporal feature of “now.” In consciousness, “now” constantly holds different contents, yet it remains “now” no matter how far it goes. This duality is thematized in Husserlian phenomenology as “the standing-streaming now.” Although this phrase appears contradictory in everyday language, it has a structure that can be clearly understood and formalized. In this paper, we show that this structure can be described as a monoid in category theory. Furthermore, monoids can be transformed into the coslice category, which corresponds to the way of perceiving present moments as juxtaposed in succession. The seemingly contradictory nature of the “now” as both flowing and standing can be precisely structured and comprehended through the monoid, while the perspective of the “now” as discrete points on a timeline can be effectively formalized using the coslice category. This framework helps us more precisely understand the differences between ordinary consciousness and meditative consciousness, specifically the experience of the “eternal now.” We illustrate how the meditative states of consciousness presented in the early Buddhist scriptures (Pali Canon) and Dōgen’s *Shōbōgenzō* remarkably reflect a monoid structure.

## 1. Introduction

“Time” has played an important role in human recognition of the self and the world, which has developed since ancient times, especially in philosophy and religion. Among the various theories of time developed in history, Edmund Husserl’s phenomenology provides a unique interpretation of how time is experienced. In the current paper, we focus on the fact that the twofold meaning of the present and the structure of time-consciousness based on it, which are discussed in Husserl’s phenomenology, seem to have a kind of mathematical structure. Given that, a certain formal expression may be possible using “category theory,” a new type of mathematics that did not exist in Husserl’s time. This paper will try to pursue this possibility. Furthermore, based on such a mathematical, category-theoretic interpretation of time-consciousness, we will show that the Buddhist view of reality based on meditative experiences is also very compatible with a category-theoretic approach to structuring temporal reality.

The aim of this paper is not merely to present a new interpretation of Husserl’s theory of time-consciousness, but to provide a phenomenological understanding and mathematical formalization of time experience itself. We will pursue this objective with the support of analyses of time and temporalization from Husserl, Kitarō Nishida, and Dōgen. One possible implication of our framework would be to offer insights into comprehending standard and altered experiences of time.

## 2. The twofold meaning of the present

In phenomenology, time has been an important topic of investigation. The phenomenological approach to time is characterized by its concern with time as lived through (experienced) subjectively, as distinguished from objective time (Husserl, 1992). We are particularly interested in the concept of “standing-streaming present” (*stehend-strömende Gegenwart*), which is discussed in Husserl’s late theory of time (Held, 1966; Husserl, 2002, 2006; Kortooms, 2002). According to it, the present has a twofold meaning.

**Figure 1:**
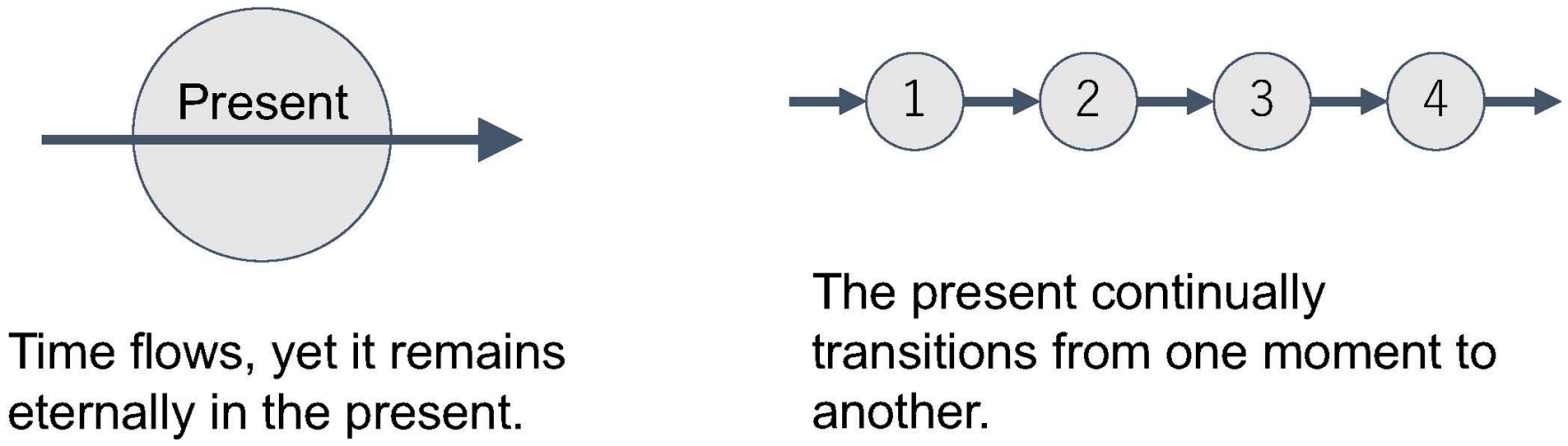
The twofold meaning of the present.

(1) On the one hand, what we experience is always “now.” Whatever we experience, it is always experienced in the present. As St. Augustine said, the past is experienced as memory in the present and the future as expectation in the present (Augustine, 2006). Husserl refers to the present in this sense as the “nunc stans” or the “standing ‘present,’” but he notes that the term “present” is not entirely appropriate here, as it usually pertains to a time modality (a single moment within the structure of “past, present, and future”), which is constituted in the “present” in the former sense (Husserl, 2002, p. 384).

(2) On the other hand, each “now” is different. What you experience “now” will soon pass away and the next “now” will appear. You might think that these are merely different *contents* passing through the same *form* of “now.” But what has passed retains the meaning of the “now that was once experienced.” If “now” is reduced to only one form, then it becomes impossible to distinguish between separate nows, such as “the former now,” “the actual now,” and “the coming now.” The fact that we can refer to different nows demonstrates that the concept of “now” cannot be reduced to a single form, but consists of multiple moments that are inseparable from the content of each now. Time can be represented as such a continuous transition from moment to moment (Figure 1).

In this way, the concept of “now” or “the present” is established in a way that cannot be reduced to a single moment or a multitude of moments, but possesses both characteristics. In other words, the present is passing, yet not passing; it is different, yet remains the same. Saying it in this way, it sounds like we are simply abusing contradictory expressions. However, the twofold meaning of the present here has a rather clear structure and can be expressed without contradiction if the appropriate method is used. In fact, in our everyday life, we accept the above-mentioned twofold meaning of the present quite naturally without question. It is included in our obvious understanding of time without any sense of discomfort.

Such a very basic structure of experience, so basic and obvious that we do not usually need to mention it, often sounds contradictory when expressed in natural language (which is why philosophy often seems to play with contradictions to those who do not share the understanding of the issues with philosophers). To describe such very basic structures, category theory, which formalizes basic structures in general, seems to be more suitable than natural language (at least in particular cases). In the following, we will attempt to express the above-mentioned twofold meaning of the present using category theory, in particular, the “monoid” and the “coslice category.”

## 3. Category and monoid

### 3.1. Category

Let us briefly explain the notion of category.^1^

A category is a system consisting of what are called “objects” and “morphisms,” and it satisfies the following conditions, which are called the axioms of category theory. In the following, we will describe each condition and then provide an intuitive explanation, followed by remarks.

**Condition 1.** For each morphism, an object called its “domain” and an object called its “codomain” are determined.

**Figure 2:**
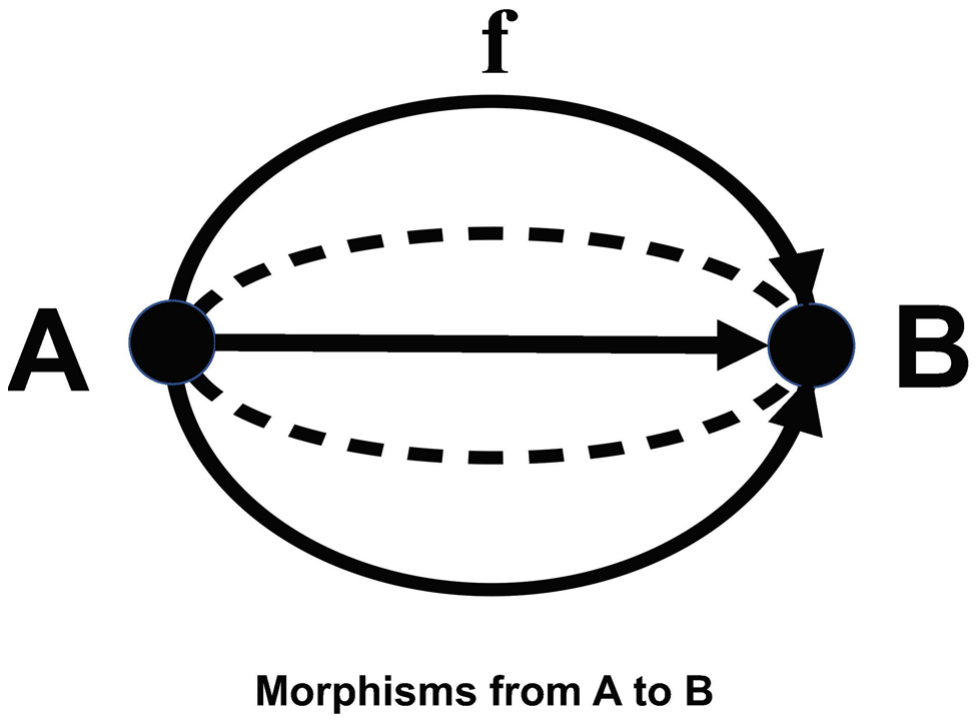
Morphisms.

**Intuitive explanation.** For intuitive understanding, it is recommended to interpret objects as “things,” “events” or “phenomena,” and morphisms as oriented “relationships,” “processes” or “transformations” between objects. Consistent with this intuition, when the domain of a morphism f is A and its codomain is B, we write f as a morphism from A to B, and say that f is a “morphism from A to B.” Note that there may be many other “morphisms from A to B” other than f (Figure 2).

**Figure 3:**
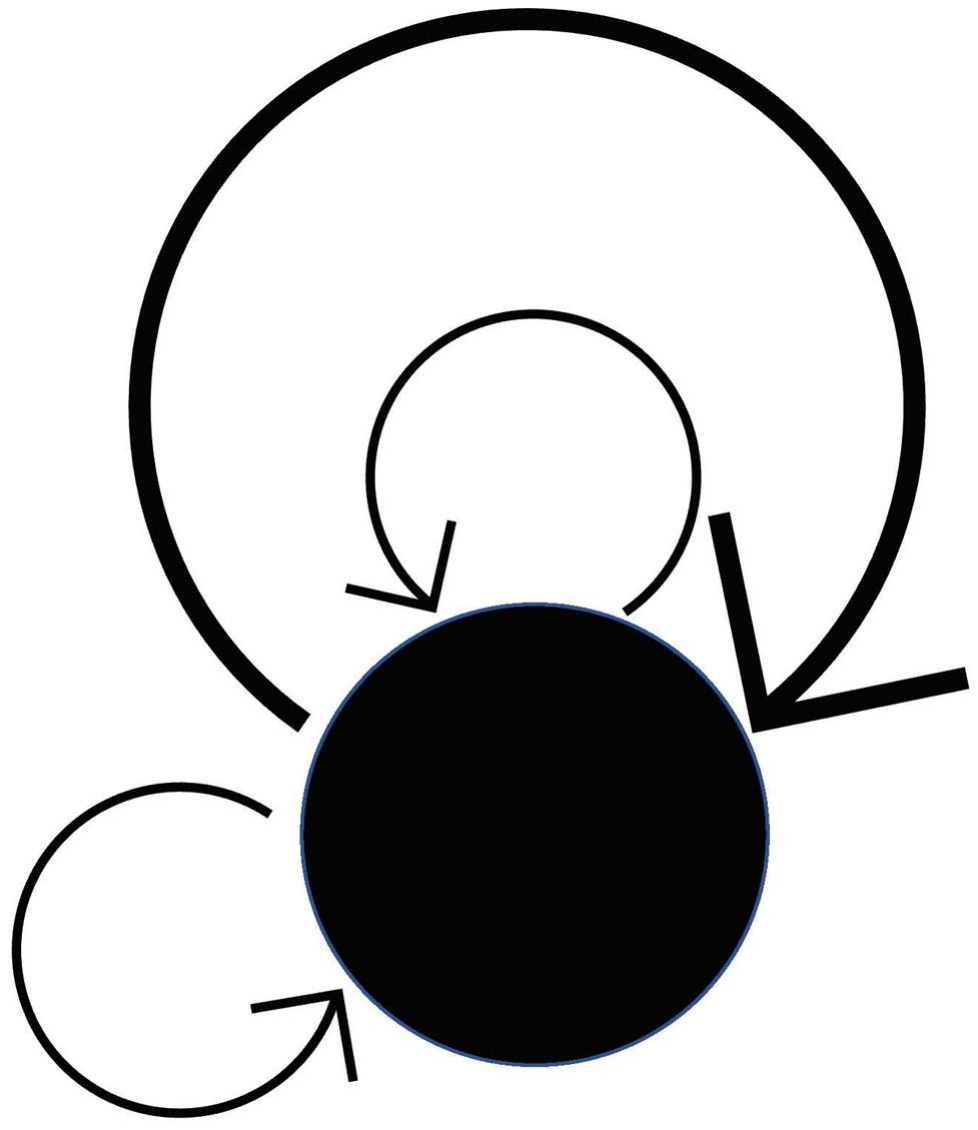
Endomorphisms.

**Remark.** However, there is no need to be bound by the above intuition (because anything that satisfies the axioms is a category). A system that satisfies Condition 1 (when the “whole” of objects and morphisms is a “set” in axiomatic set theory) is called a “directed graph.” For general directed graphs that are not categories, the terms “vertex,” “edge,” “source” and “target” are used instead of the terms “object,” “morphism,” “domain” and “codomain.” Note that the domain and the codomain may coincide. A morphism in which the domain and the codomain coincide (like a “loop”) is called an endomorphism (there can be many endomorphisms) (Figure 3).

**Figure 4:**
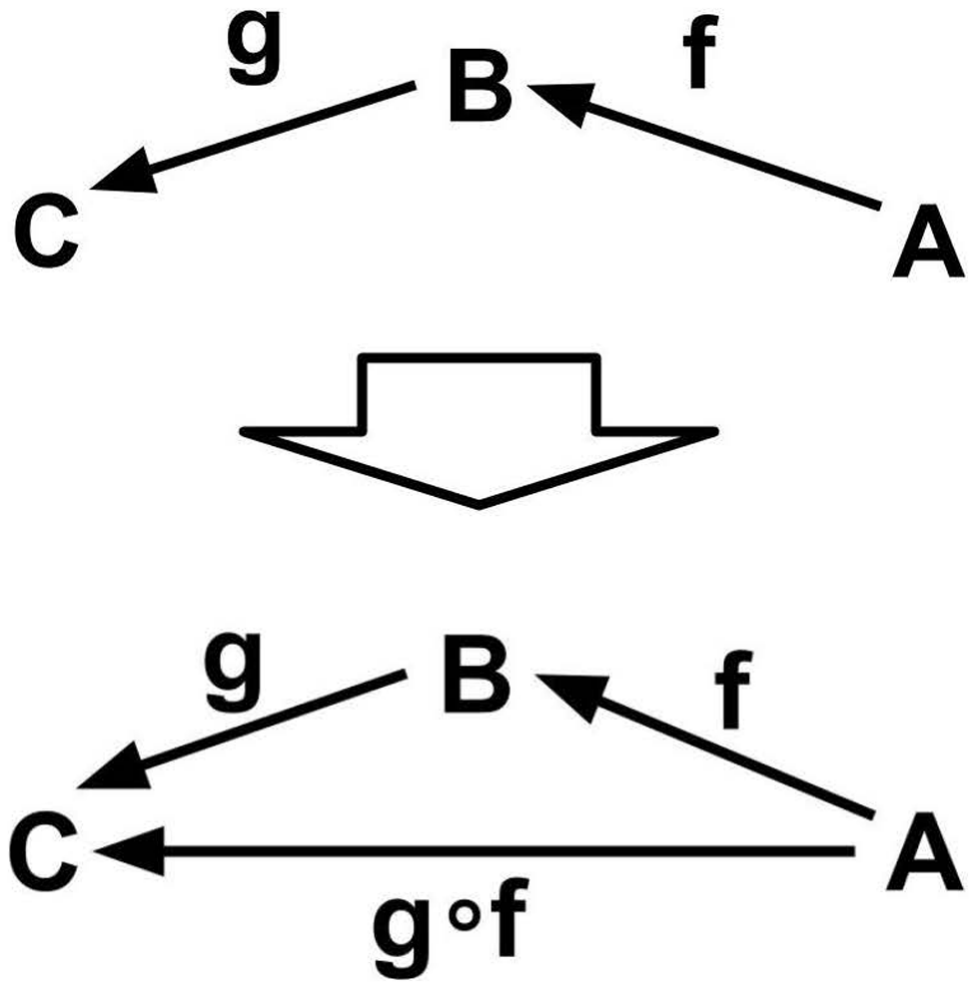
Composition.

**Condition 2.** An (ordered) pair (g,f) of morphisms is said to be composable if the codomain of the morphism f coincides with the domain of the morphism g. For a composable pair of morphisms (g,f), there is an operation ∘ called “composition” that makes the morphism g∘f called the “composite of f and g.” The domain of g∘f coincides with the domain of f, and the codomain of g∘f coincides with the codomain of g (Figure 4).

**Intuitive explanation.** For example, if f and g are “processes” and the “final state” (codomain) of f and the “initial state” (domain) of g are the same, we can think of the “composite” g∘f as a “connected process,” and the operation “composition” as the operation of “connecting” processes (morphisms).

**Remark**. Note that if the codomain of one morphism does not match the domain of the other morphism, the composite morphism is not defined. Composition of any pair of two morphisms is always possible only when there is only one object, because only in this case, the domains and the codomains of the morphisms are the same without exception. A category with only one object is called “monoid” (when the “whole” of objects and morphisms is a “set” in axiomatic set theory), which we will focus on in the next subsection and which will play a crucial role in the paper.

**Condition 3 (Associative law).** For any morphism f,g,h such that (h,g) and (g,f) are composable, (h∘g)∘f = h∘(g∘f) holds.

**Intuitive explanation**. In short, “the order of parentheses does not matter.” If we think of morphism as a “process” and composition as an operation that “connects processes” (just connects them without doing anything additional), it is a condition that naturally seems to hold.

**Remark.** However, general operations do not always satisfy the above condition (“associative law”). For example, (5 + 3) + 2 = 5 + (3 + 2) holds but (5−3)−2 = 5−(3−2) does not. It can also be seen that the associative law demands that composition be a “fairly simple type of operation,” such as “just connecting processes.”

**Figure 5:**
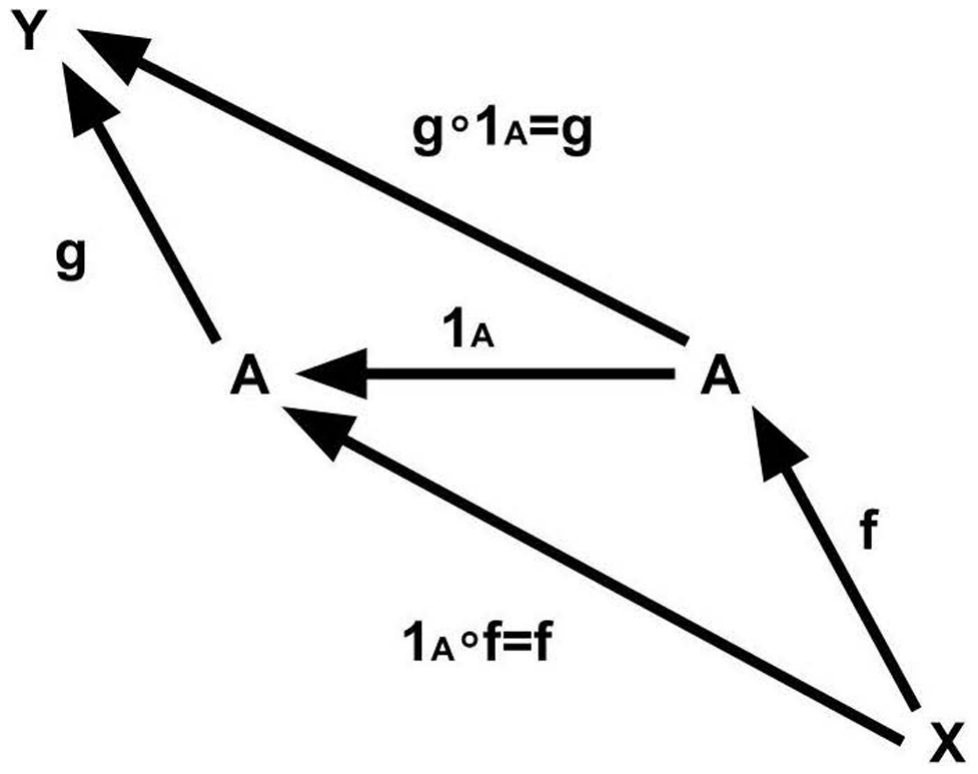
Identity morphism.

**Condition 4 (Unit law).** For each object A, there exists a unique morphism 1_A_ whose domain and codomain is A which satisfies “1_A_∘f = f for any morphism f whose codomain is A” and “g∘1_A_ = g for any morphism g whose domain is A.” Morphism 1_A_ is called the “identity morphism” of A (Figure 5).

**Figure 6:**
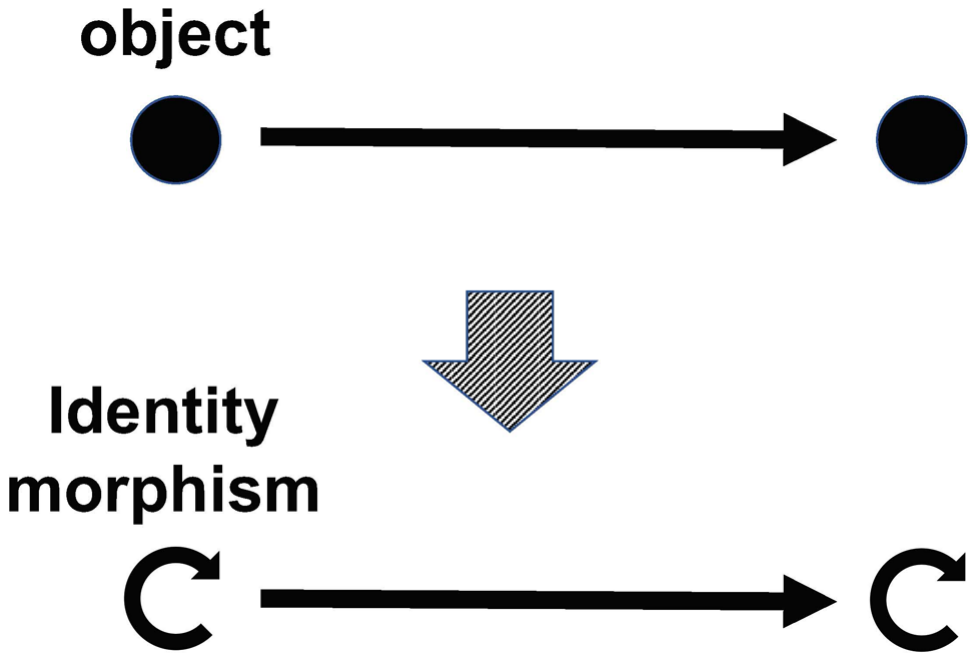
Object = identity morphism.

**Intuitive explanation**. Each object corresponds to an identity morphism, which is a morphism that “does nothing” (a morphism that plays the role of “1” in multiplication). Due to this condition, we can “identify” each object with its identity morphism (Figure 6). In other words, this condition makes it possible to think of objects as just a special kind of morphism.

**Remark.** The part of condition 4, “unique” is actually unnecessary. This is because if it is guaranteed that the identity morphism exists, the “uniqueness” of such a morphism for each object holds automatically. (To prove this is a good exercise of category theory.) Note that although there is only one identity morphism for each object, there can be an infinite number of endomorphisms.

### 3.2. Monoid

**Figure 7:**
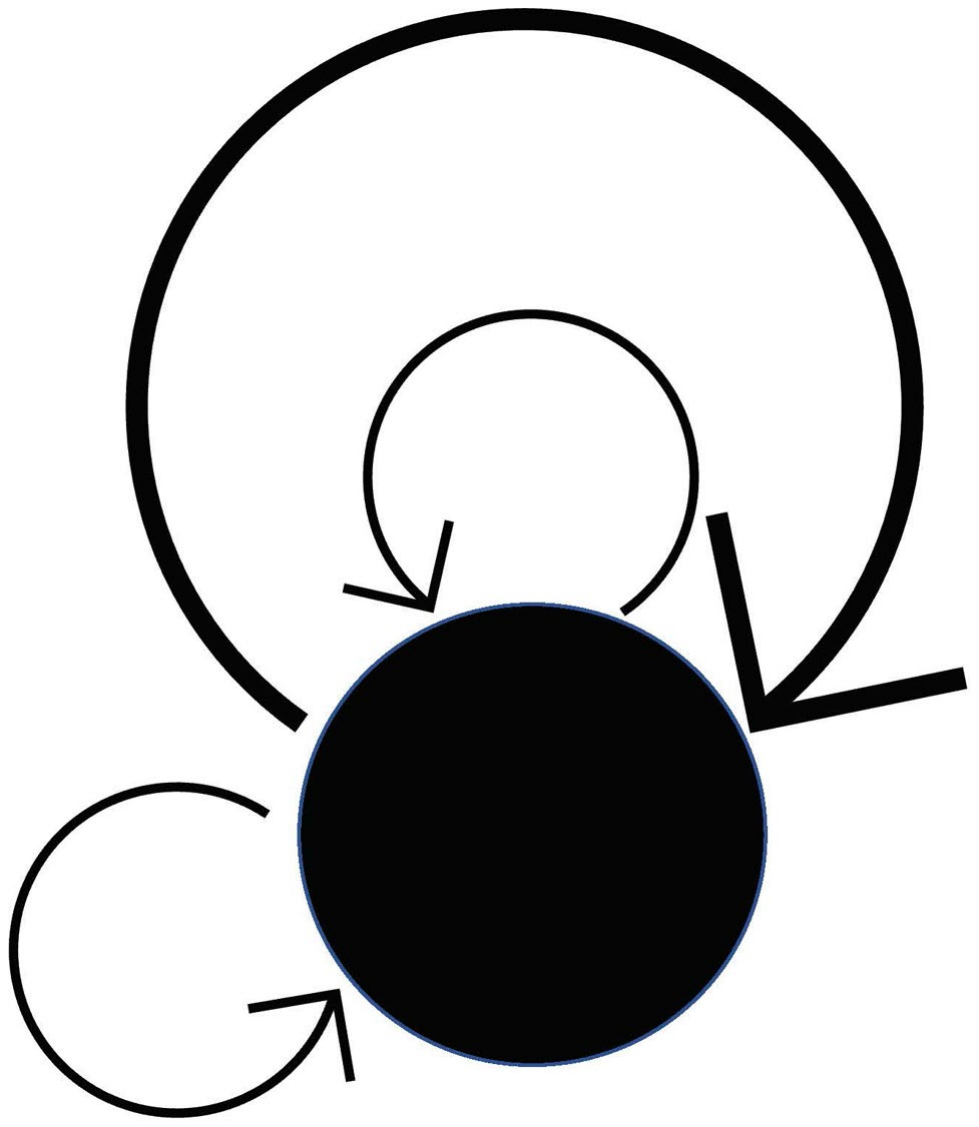
Monoid.

A category with only one object is called a monoid. (See also the remark for condition 2 in the axioms of category theory). Since there is only one object, any two morphisms of a monoid are composable. In other words, composition becomes a “dyadic operation” of morphisms. (Note that every morphism in a monoid is an endomorphism by definition, as it is easy to see from Figure 7^2^).

As a simple example, let us define a monoid whose unique object is a point p in the space E and whose morphisms are all the paths (loops) in the space E from p to p. Although point p itself has no structure, the monoid defined above contains rich structures and captures the information of the structures of E viewed from the perspective of point p.

Let us take another example. The set of all natural numbers can be thought of as a monoid where every natural number is a morphism, the number 0 is the only object (recall that an object can be identified with the identity morphism), and composition is addition.

Note also that any group is a monoid. The concept of monoid is a generalization of the concept of group. Readers may find it interesting to try to give other examples.

## 4. Monoid and the structure of time

Now, we are ready to interpret the structure of time in terms of category theory. In this section, we will demonstrate that this idea is compelling.

The monoid is a very primitive structure and can be found in many places. The examples in mathematics are too numerous to list, and in the real world, as well, there are often structures that develop and constantly return to the same state, which have a monoid structure. Time is one such structure. Time consists of processes, whose beginning is the present, and whose end is also the present. Thus, time can be said to have monoid-like characteristics, since the processes in time are always “processes from now to now.”

**Figure 8:**
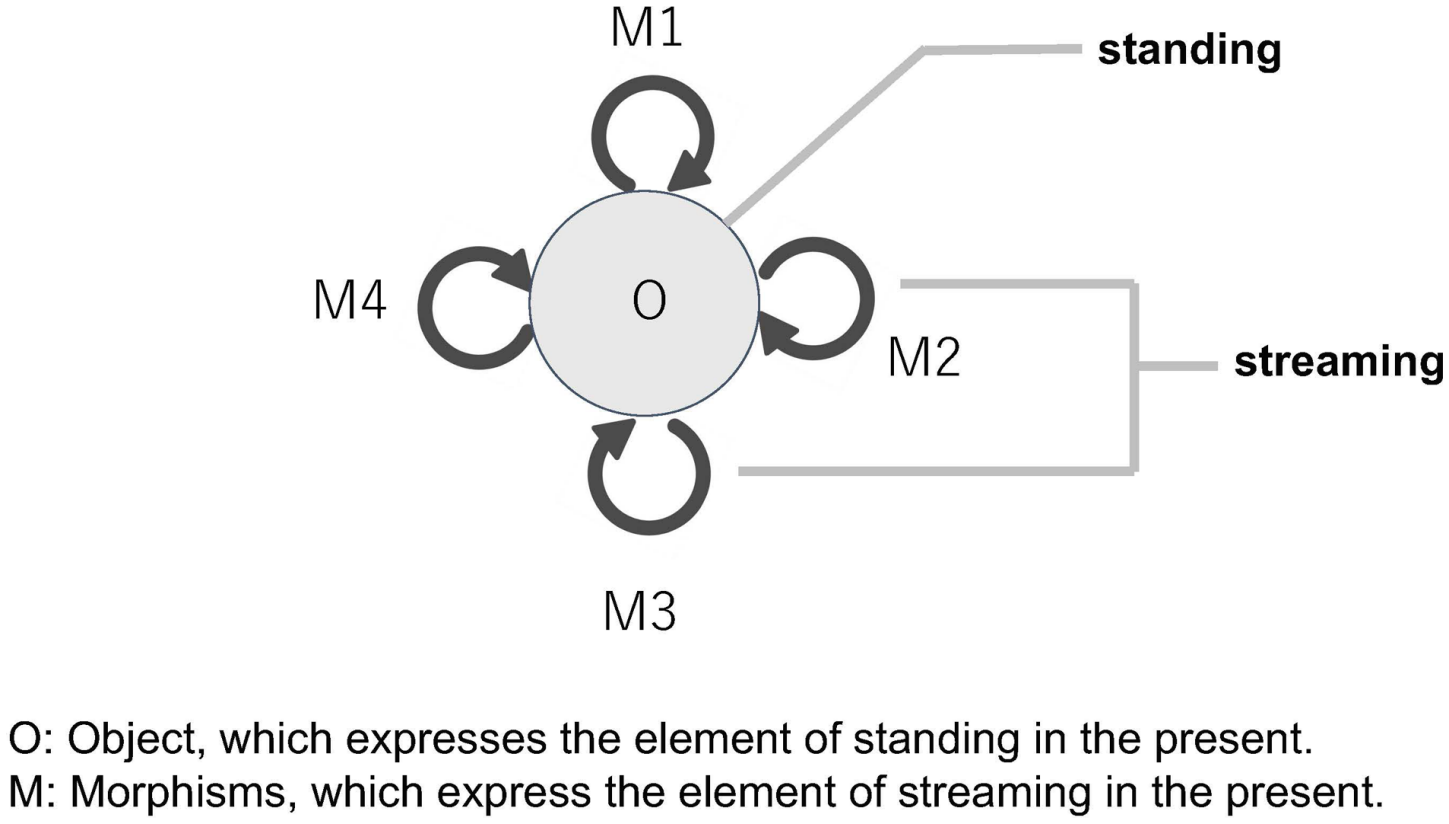
Representation of the standing and streaming aspects of the present within a monoid structure.

We pointed out with Husserl that the present in time has two different characters simultaneously: the standing and the streaming. The monoid beautifully formalizes this structure. A monoid consists of a number of morphisms, all of which represent the flow of time. The continuous unfolding (or the extension) of the present can be expressed by these morphisms. On the other hand, all morphisms of a monoid come from the same object as its domain and end up in it as its codomain, which can represent that temporal flows are perpetually standing in the same place (*nunc stans*). In short, the streaming aspect of the present corresponds to the various morphisms of a monoid, whereas the standing aspect corresponds to the unique object of the monoid. In this way, a monoid effectively captures the structure of the present, encompassing both its standing and streaming aspects (Figure 8).

Time has also the structure of *modification*, in which the “present” that has already flowed away can be experienced as “being past.” The “past present” is a temporally modified present. How is this structure represented in a monoid? We can focus on the *composition* of morphisms. Given that the morphisms of a monoid are composable, and that the result of such composition is also a morphism of the same monoid, the structure that preserves multiple pasts in the same standing “present” is implied in the monoid structure.

Some may argue that the structure of time does not align with the structure of the monoid because each present is a different present (each present is distinct from the others). However, when we think of each present as a “different present,” this view is already a result of certain objectification, abstraction, and spatialization. In such cases, we look at the present not from the standpoint of “living through” it, but rather as if multiple presents were lined up side by side in front of us. Of course, this view is not inappropriate in all respects, but it is a higher-order view derived from the most basic view of the present. This derivative view will be discussed later in this paper using the term “coslice category.” In contrast, the most basic structure of time is considered to be a monoid-like one, i.e., consisting of processes that constantly move from the same to the same.

We may further respond to the above objection as follows. Suppose time is made up of different presents. In this case, it becomes a difficult problem to explain how these different presents can be integrated into a single time. However, in fact, time is naturally united as a single stream. On the other hand, if there were only one static present, there would be no time. Therefore, it is natural to think of time as a process, but simultaneously as “coming back to the same place” in some way. At least when we consider experiential time, we cannot help but say so because, as we already said, time, in its constant motion, remains both the same and different simultaneously.

Next, we can further support our interpretation by noting that the descriptions of time by some philosophers naturally remind the nature of the monoid. Husserl attempted to conceive of time with such a primitive concept. The concept of “standing-streaming present,” which we have already mentioned, seems to express precisely the monoid-like “process of continually returning to the same place.” Kitarō Nishida, a modern Japanese philosopher of the Kyoto School, likewise speaks of the “eternal now,” which also expresses the monoid-like nature of time as a process of continually returning to the present (Nishida, 1948, p. 181–232). While these expressions may appear inherently “contradictory” in everyday language, it is possible to consider that both Husserl and Nishida had a highly clear structure in mind. When attempting to express these ideas in natural language, however, the resulting expressions inevitably appear “contradictory” and even mysterious.

In the time of Husserl and Nishida, of course, category theory did not yet exist. However, Nishida attempted to express the structure of the self using the concept of “group,” which today is considered a special case of monoids,^3^ and it is quite possible that what he was describing in his “contradictory” expressions was based on intuitions that could be called mathematical. (Nishida originally attempted to become a mathematician.) Husserl started out as a mathematician, and his arguments often contain statements that suggest a mathematical structure. If category theory had existed during the time of Husserl and Nishida, they might have readily embraced the idea.

For example, the following statement by Nishida may seem less paradoxical when read with the idea of monoid structure in mind.

Time disappears everywhere and is born everywhere as the self-limitation of the eternal now. Therefore, time touches the eternal now at each moment. It can be said that time disappears moment by moment and is born moment by moment. Time can be thought of as a continuity of discontinuity. (Nishida, 1948, p. 342; our translation)

At first glance, the statement “time touches the eternal now at each moment” may sound mystical. However, when we consider the monoid’s characteristic of “always coming back to the same no matter what operation is performed,” the former statement seems to convey essentially the same idea as the latter. There is nothing mysterious or contradictory about the “continuity of discontinuity” if we think of it in terms of the structure of the monoid, in which each morphism of the monoid is a different one, but all are connected through the same object. What Nishida was trying to say may have been something very simple but difficult to express in everyday language, as is often the case with the monoid concept.

Finally, we would like to emphasize the significance of formalizing the structure of experience, particularly that of time-consciousness, using category theory. A monoid is mathematically very simply defined, but it sounds contradictory in some cases. As examples, we can consider statements such as “there can be an infinite number of morphisms from one object to itself,” or “every morphism goes from the same to the same, but they are all different.” If these expressions seem strange, it is likely because when the morphism is understood as a “relation,” people tend to think that there is only one morphism from object X to object Y. The notion of “relation” is often thought to be reducible to a pair of relata. (Even in mathematics, a relation on a set is defined as the set of pairs of relata belonging to the set.) However, the morphism in category theory is not of the kind that can be reduced to such pairs of relata. Therefore, of course, there can be innumerable (or sometimes no) morphisms from X to Y. In particular, there are innumerable morphisms from X to X. By definition, there are identity morphisms, but there can be any number of other morphisms. This diversity is the source of the mathematical interest in the concept of a monoid.

In short, what is being revealed here is something richer than the concept of a mere “relation,” which cannot be simply reduced to a mere “pair of relata,” and this something may indeed be closer to our actual experience.^4^ It is not that the monoid in category theory coincides with the structure of our experience of time by chance, but it is because the morphism of category theory expresses a rich dimension that falls outside the scope of the simple concept of relation, as we have just described. It matches the very basic structures of experience and reality, which normally can only be expressed in a contradictory manner.

## 5. Modification: from monoid to coslice category

**Figure 9:**
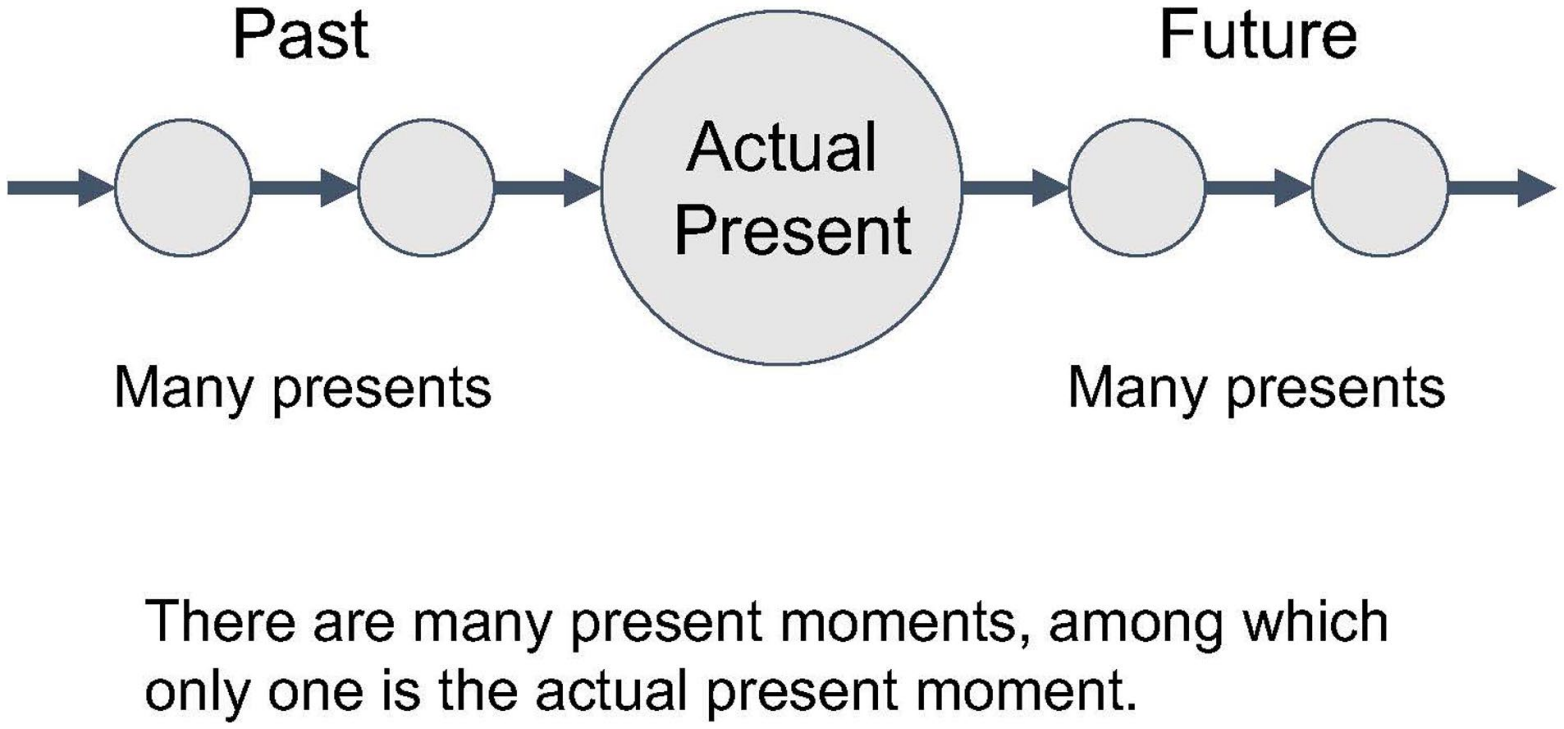
The actual present within a multitude of present moments.

In the next step, we will focus on another aspect of the experience of time and try to formalize it using another type of category: Coslice category. Different from the “monoid perspective” of time, which represents the fundamental dimension of time experience that is hard to describe, we can also talk about time in a more “rationalized” manner. Fundamentally (or, from a monoid point of view), we can say that time consists of a present that is different in each moment and always the same. On the other hand, however, we can also consider each present as a separate object, and among such “many presents,” the “actual present moment” as a special present. In this case, unlike when viewed from a monoid perspective, there is no particular contradiction, since the present is being viewed as a “multiplicity of objects” (Figure 9).

In Husserl’s phenomenology, such a view of time is described in terms of *modification*. The term “modification” is also used in a very basic and universal sense; for example, the present moment (primal impression) is “retentionally modified” and loses its living actuality. It transitions into the immediate past. This continuous transition is called “retentional modification” (Husserl, 1992). We can assume that this corresponds to morphisms and their compositions in category theory. However, what we would like to focus on here is a more global structural modification. That is, the view of the present moments as a series of distinct objects is already different from the way we experience time in the midst of the actual time experience itself. We do not see the ever-changing present as individual (discrete) objects in the latter case. It is always experienced as the same present, even though it is constantly changing. Therefore, we can speak of a “modification” from such a more fundamental experience of time to an experience that objectifies time, in which the mode of experience changes.

Let us consider how to formalize this “modified” experience of time using category theory. In this type of time experience, multiple presents are differentiated and juxtaposed. This differentiation corresponds to the fact that the various morphisms of a monoid are differentiated. In other words, the individual presents, observed in juxtaposition in the “modified” experience, can be viewed as objectified forms of the various morphisms of the monoid. Here we need to focus on a certain construction that plays an important role in category theory: the construction that creates a new category by focusing on a certain object of the original category and seeing the morphisms from it as objects. This new category is called the coslice category. The operation described above called “modification” can be expressed as an operation to construct a coslice category from a monoid.

**Figure 10:**
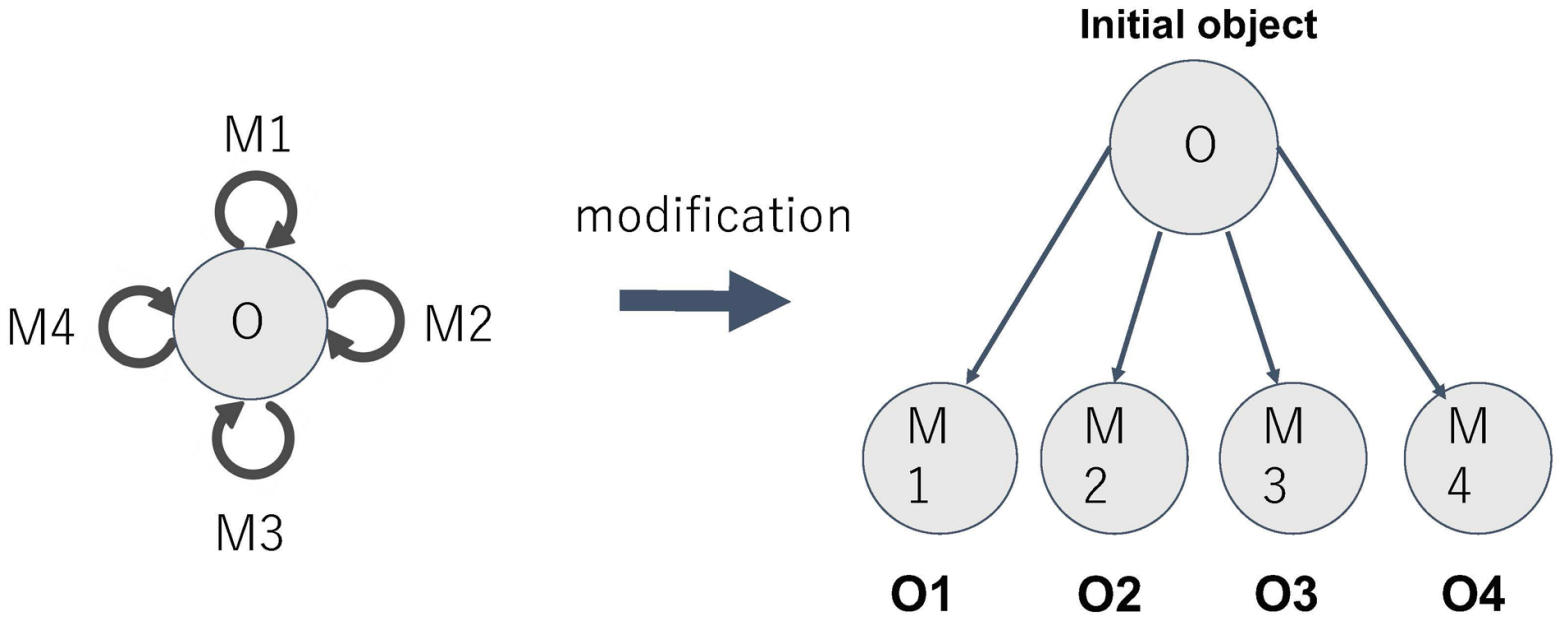
Relationship between monoid and coslice category.

As already mentioned, the monoid is a category that has only one object. On the other hand, there can be any number of morphisms. By taking those morphisms as objects, we can construct a coslice category. In this case, since the monoid has only one object, the coslice category is uniquely determined. The unique object in the monoid can also be regarded as a special type of morphism, i.e., the “identity morphism,” and thus, it is also included in the coslice category as an object. This becomes a special type of object, called an “initial object,” in the coslice category (Figure 10).

Returning to the discussion about time, the unique object of the monoid corresponds to the present when we say that “the present is always present.” On the other hand, the various objects in the coslice category correspond to the presents viewed as juxtaposed, and the initial object of the coslice category corresponds to the objectification of the “actual present currently being experienced.” Although this objectified “actual present” still retains a special meaning, it is merely *a present* taken in juxtaposition with various other presents. (In this sense, it differs from the standing “present,” which is conceived as the unique object of the monoid, i.e., the identity morphism.) This view fits very well to the formalization of the modified time experience through coslice category.

Let us quote some passages from Husserl’s late research manuscripts to further explore the meaning of the modification from the fundamental to the objectified view of time. Husserl speaks of “standing-streaming self-present (*Selbstgegenwart*)” as the “primal phenomenon in which everything else that may be called phenomenon in any sense has its source” (Husserl, 2006, p. 145). He also talks about “the sphere of the primal temporalization, in which the first and primally welling meaning of time appears—time just as living streaming present. All other temporality, whether subjective or objective—whatever sense these words may have—receives its sense of being and its validity from it” (Husserl, 2002, p. 187; see also Husserl, 2006, p. 1–3, 40).^5^ Here, Husserl refers to the living present as a “source” (or “wellspring”). However, the relationship between the “source” and “what springs forth from it” is not immediately obvious. Husserl also speaks of the living present as the “primal mode of the present.” This indicates that there is the primal and the derivative mode of the present. It seems possible to think of the relationship between the “primal source” and “what springs forth from it” as the relationship between the “monoid-like present” and the “various objectified and juxtaposed presents.” At the very least, we may assume that “what flowed out from the source” suggests a linear view of time, portraying various presents as standing side by side in succession.

Such a development from the “primal mode of the present” to the “multiple presents” can be represented as a development from the monoid to the coslice category. If we can comprehend the primal present and the sequentially juxtaposed presents as being in a source-derivative relationship, it would be appropriate to formalize their relationship through the construction of the coslice category from the monoid. The structure of the original monoid is faithfully preserved in the coslice category, which reflects the character of modification in the constitution of juxtaposed presents. Simultaneously, the uniqueness of the primal present and the multiplicity of the modified presents are well represented by the unique object of the monoid and the multiple objects of the coslice category.

## 6. Meditation: from coslice category to monoid

If we can understand the experience of time as we have described, it becomes possible to use this framework to illustrate various transformations of time experience. For example, it is often reported that time experience is transformed by mental illnesses such as schizophrenia and depersonalization, by taking psychedelic drugs such as LSD, and by meditation such as mindfulness (Novak, 1996; Kramer et al., 2013; Wittmann and Schmidt, 2014; Wittmann et al., 2015; Linares Gutiérrez et al., 2022). Here, as an example of the experience gained through meditation, we will take up the Buddhist recognition of time as an expression of the experience obtained through Buddhist meditation practice (*zazen*), and discuss how it can be interpreted in terms of our framework.

Gautama Buddha says that the following things are what Buddhist practitioners should keep in mind.

The past should not be followed after, the future not desired.

What is past is got rid of and the future has not come.

But whoever has vision now here, now there, of a present thing,

Knowing that it is immovable, unshakable, let him cultivate it. (Horner, 1999, p. 233)

Here, it is stated as a training principle for Buddhist practitioners to concentrate on the “present” rather than focusing on the past or future. This may imply a return from the coslice category-like view of time, in which the various presents are mutually juxtaposed side by side, to the monoid-like view of time, which consists of only one object and various morphisms.

Dōgen, a Japanese Zen master, provides a more structured description of this idea in his work *Shōbōgenzō*. Dōgen coined the term *Uji* (being-time), suggesting that what is called “being” is, in fact, time (Dōgen, 1990). In terms of category theory, various “beings” in the usual sense can be interpreted as the objects of the coslice category because they are distinct individual things. However, if we bring the coslice category back to the original monoid, that is, if we reduce it to the original “time” (or temporalization), “beings” are in fact morphisms. In the monoid, all the morphisms pass through only one point, the unique object of the monoid. If we take the point of view of the unique object of the monoid, it leads to all the morphisms. Of course, we cannot see all the morphisms, but we can notice that all the morphisms pass through this one point.

Viewing each now as a discrete point in time means objectifying the now (seeing it as a being). This corresponds to a coslice category whose objects are the respective now as morphisms. On the other hand, if we convert the coslice category to its original category, there is only one unique object which is the domain and the codomain of all morphisms. At this unique object, all the morphisms are connected. If we stand on the object of this monoid, we can obtain a picture in which there is always an invariant among the innumerable morphisms, and all of them are connected at this invariant point.

This is just like the view that “time is always flowing and moving, yet at the same time it is still.” It is close to the Buddha’s view that there is only now.

In meditation practice, it is said that we should focus on the “now,” and the Buddhist perception of reality gained through meditation emphasizes the perspective of seeing everything in the “now,” but this does not mean that we simply cut off the past and future and leave only the present among the various points in time (past, present, and future). Rather, it is essential to realize that there is a structure in our experience that allows us to go through the “now” to all of time. In our view, this means becoming aware of the monoid structure in our experience of time and (re)activating it consciously. When Buddhist practitioners talk about “focusing on the now,” what they have actually in mind is not the separate moment of the “now” but the monoid structure, which is composed of numerous becomings (morphisms) and designates the “now” as its unique object.

Dōgen says, “…it may look like it is far away in the distance, but it is the Now…” (Dōgen, 1990: the chapter called *Uji*).^6^ This means the one and only now, which can also be called the eternal now. He further structures this statement as follows.

“If time is not seen as flowing, then the time when you climb the mountain and look around is the Now of the being-time. Even if time is seen as flowing, there is the Now of the being-time for me. Thus, the time seen as flowing is also the being-time.” (Dōgen, 1990).

Time is always now, whether we see it as flowing or not. If time has a monoid structure, it is also possible to view all morphisms from the perspective of the object of the monoid. This means that all time can be viewed from the eternal Now that corresponds to the unique object of the monoid. In this view, our perspective is on the object of the monoid, which never changes, and in terms of time, we are always in the “now.”

On the other hand, we can also trace each morphism of the monoid. This view corresponds to the view that time is always flowing. Even if we take this view, since each morphism is always from one and the same object to the same object, we can say that we are always in the “now” in spite of the passage of time.

Monoid-like structures are also frequently found elsewhere in Dōgen’s text (the *Uji* chapter).

“Being-time has the virtue of passage. That is to say, today passes to tomorrow; today passes to yesterday; yesterday passes to today; today passes to today; tomorrow passes to tomorrow. This is because the passage is the virtue of time.” (Dōgen, 1990)

One morphism and another, one morphism and itself, can all be composed because the morphism of a monoid has only one object, which means that all morphisms (“passages”) are connected at the same object (“now”) because their starting points (domains) and endpoints (codomains) are one and the same object (“the eternal now”).

One of the essential points of *zazen* (zen meditation) is to become aware of this kind of structure, the monoid structure of time. The monoid structure of time usually recedes into the background in our consciousness, and the dominant structure in our consciousness is that of the coslice category. In contrast, in *zazen* meditation, it is desirable to return to a monoid view of time, in which all time is connected in the “here and now” and is simultaneously present, rather than the coslice category view of time in which each point in time is viewed in succession.

Moreover, if we also see that the coslice category view of time can be generated from the monoid view of time by certain consistent operations, then the view of time that we usually think of as consisting of separate points in time can be seen simultaneously from a monoid perspective. This makes it possible for all time to be seen in the only temporal structure in which all time is Now, and all beings as temporally existent.

By viewing time from the perspective of the monoid/coslice category, as described above, it is possible to make consistent interpretations of the experience of time as it is talked about in the context of the meditation experience. At the very least, we can say that beyond the naive view, it offers a more structured view of what it means to focus on the now. In other words, we propose that focusing on the now does not simply mean focusing on the now as one of a series of juxtaposed points in time, but that it means looking at all events as a myriad of morphisms that arise from the now and becoming aware of the monoid structure of time as such.

This is only one implication of our framework. Starting from our framework, it may be possible to interpret the altered temporal experiences seen in depression, depersonalization, schizophrenia, autism, and so on. We plan to cover this subject matter in a different article.

## 7. Conclusion

In this paper, starting from a phenomenological theory of time, we interpreted the structure of time-consciousness by using the structure of the monoid and the coslice category in category theory. This allows us to unite, without any contradictions, the view of the various “nows” placed side by side and the view that “what we are experiencing is the now, no matter how far our experience goes.” The structure of the temporal present is often described in contradictory terms such as “standing still while flowing,” or “moving and still.” Our framework makes it possible to fully grasp the intent of these natural language expressions and to bring them into a consistent understanding. This does not mean that we merely offered a convenient method of abstraction to explain the structure of time experience. Instead, what we presented was a phenomenological and formal expression of time (or temporalization) itself, as experienced both primally and derivatively.

Such an interpretation of time using category theory fits nicely into the Buddhist description of the meditative experience. In meditation, “focusing on the now” does not imply a dismissal of any point in time other than the now, but rather a reduction of the view of time to a monoid view, suggesting a position of seeing everything from the now (the only object of the monoid). Such a view is a natural outgrowth of our framework.

In sum, the monoid provides us with a simple yet content-rich structure. Our experience contains numerous structures that may seem contradictory when expressed in natural language. However, category theory allows us to understand such structures with great clarity. This paper illustrates these possibilities of category theory by examining time as its subject matter.

## Data availability statement

The original contributions presented in the study are included in the article/supplementary material, further inquiries can be directed to the corresponding author.

## Author contributions

ST and HS conducted the entire process of completing the work. All authors contributed to the article and approved the submitted version.

## Funding

This work was supported by the JSPS KAKENHI (Grant Nos. JP 20H00001 and JP 23H04829).

## Conflict of interest

The authors declare that the research was conducted in the absence of any commercial or financial relationships that could be construed as a potential conflict of interest.

## Publisher’s note

All claims expressed in this article are solely those of the authors and do not necessarily represent those of their affiliated organizations, or those of the publisher, the editors and the reviewers. Any product that may be evaluated in this article, or claim that may be made by its manufacturer, is not guaranteed or endorsed by the publisher.

## Footnotes

^1^The explanation of this section is based on Fuyama and Saigo (2022).

^2^This is the same as Figure 3, but since monoid consists of only one object and endomorphisms, it can be illustrated by the same figure.

^3^At that time, the concept of monoids had not been established, and the formulation of groups was more complex because category theory did not exist.

^4^Taguchi (2019) describes the rich reality beyond such relations as “mediation.” “Mediation” is more than relation, and the Japanese philosopher Hajime Tanabe describes it as “connecting by cutting” (Tanabe, 1963a, p. 335, Tanabe, 1963b, p. 486). In other words, “mediation” is a concept that includes both difference/disconnection and connection/relationship, and reality is considered to consist of such “mediations” in the most basic sense.

^5^All English citations from Husserl’s German writings are translated by us.

^6^Our English translations of Dōgen’s texts are based on the modern Japanese translation by Yorizumi (2015).
